# Increase of glutathione, testosterone and antioxidant effects of *Jurenia dolomiaea* on CCl_4_ induced testicular toxicity in rat

**DOI:** 10.1186/s12906-017-1718-z

**Published:** 2017-04-08

**Authors:** Naseer Ali Shah, Muhammad Rashid Khan

**Affiliations:** 1 Department of Biosciences, COMSATS Institute of Information Technology, Islamabad, Pakistan; 2 Department of Biochemistry, Faculty of Biological Sciences, Quaid-i-Azam University, Islamabad, 45320 Pakistan

**Keywords:** *Jurenia dolomiaea*, Antioxidant enzymes, Lipid peroxides, Gsh, Testosterone

## Abstract

**Background:**

Root of *Jurenia dolomiaea* is used traditionally in various disorders involving oxidative injuries i.e. rheumatism, gout and as stimulant. Earlier we have investigated in vitro antioxidant and DNA protective ability. In this investigation we have evaluated protective potential of *J. dolomiaea* root against the oxidative injuries induced with carbon tetrachloride (CCl_4_) in testes of rat.

**Methods:**

Dried roots of *J. dolomiaea* were powdered and extracted with 95% methanol and residue was fractionated in escalating polarity of solvents. On the basis of potent antioxidant ability; the ethyl acetate fraction (JDEE) was selected to evaluate the in vivo antioxidant activity against CCl_4_ induced oxidative stress in rat. Sprague Dawley male rats (42) were equally divided in to 7 groups: control, vehicle control, JDEE (400 mg/kg; p.o.) alone, CCl_4_ (I ml/kg; 1:10 *v*/v in olive oil) alone, JDEE (200 mg/kg, 400 mg/kg) with CCl_4_, and silymarin (200 mg/kg) with CCl_4_ on alternate days for 60 days. Testes samples were investigated for antioxidant enzymes, biochemical markers and histopathology while the serum samples were analyzed for the testosterone level.

**Results:**

Administration of CCl_4_ to rats depleted the activity level of antioxidant enzymes viz.; CAT, POD, SOD, GST, GPx, and GR, and the concentration of protein and GSH while enhanced the level of lipid peroxides (TBARS), H_2_O_2_ and nitrite in testes samples of rat. Concentration of testosterone in serum of rat decreased with CCl_4_ treatment. Co-treatment of silymarin and the JDEE (200 mg/kg, 400 mg/kg) lessened the toxic effects of CCl_4_ and reversed the level of these parameters towards the control group. An admirable increase (*P* < 0.05) in the level of GSH in testes, testosterone in serum and thickness of germinal layers in testes with JDEE (400 mg/kg) alone was recorded. Histopathological observation of testes samples endorsed the alterations induced with different treatments.

**Conclusions:**

JDEE co-treatment to rats ameliorated the toxic effects of CCl_4_ in testes samples. Enhanced level of GSH, thickness of germinal layers in testes and testosterone in serum with JDEE (400 mg/kg) treatment alone to rats demanded the evaluation of JDEE for sexual behavior.

## Background

Male infertility is a major clinical problem affecting 30% of the world population [1]. Among several factors affecting the fertility are the erectile dysfunction, premature ejaculation, quality and quantity of sperms. These effects might be caused due to the production of free radicals in testes. Clinical disorders such as hypertension and atherosclerosis which are usually induced with reactive oxygen species (ROS) also provoke the erectile dysfunction. Male sexual characteristics are regulated by androgens; hypogonadism induced with decrease of natural antioxidant capacity (glutathione) and/or increase of ROS may cause loss of libido in adults which badly affected the sexual behavior [2].

Various reports revealed that carbon tetrachloride (CCl_4_) a well-established hepatotoxin also causes injuries in testes [2, 3]. CCl_4_ is metabolized in the tissues to highly reactive trichloromethyl radical which starts free radical induced lipid peroxidation of cytoplasmic membrane phospholipids and brings pathological changes in the cell membrane by accumulation of lipid derived oxidants. Exposure of rats to CCl_4_ causes oxidative injuries in testes of rat leading to decrease the activity level of antioxidant enzymes and substances such as glutathione (GSH) while enhanced the lipid peroxidation. Histopathological investigation of testes revealed the damaging action of CCl_4_ in rat. Treatment of CCl_4_ causes hypogonadism and decrease the level of testosterone in serum of rat [3, 4]. To cope with such reactive species and the clinical disorders, it is essential to obtain dietary antioxidants which counter measure the excessive generation of free radicals [3, 4].

*Jurinea dolomiaea* Boiss (syn. *Jurinea macrocephala* Royle) has a place with Family Asteraceae (Compositae). It is a prostrate perennial herb with a thick terminal group of large flower heads and a rosette of long spreading lobed leaves with purple mid veins, root long, tuberous. It is found in scattered places in Byas and Darma valley in Northern areas of Pakistan. Root is utilized in the form of poultice as antiseptic in skin eruptions while its decoction is given in colic. Additionally, it is recognized cordial and is given in puerperal fevers. Roots are acknowledged to be immuno-stimulant and given in fever after labor [5, 6]. Roots are also used by the local population for loose bowels and stomachache [6]. Local communities use the crushed roots for skin eruptions [7, 8] while the aromatic oil obtained from roots is used in gout and rheumatism [9]. Ahmad and Habib [10] reported that local communities use the extract of roots as tonic for weakness of the bones. Roots are cooked with maize flour and used for the treatment of bone fractures. Antimicrobial activity of the leaf extracts of *J. dolomiaea* have been reported by Dwivedi and Wagay [11]. Antioxidant and antibacterial activities of the *J. dolomiaea* plant have also been evaluated [12]. Earlier we have reported in vitro antioxidant and DNA protection ability of the methanol extract and its derived fractions. Among the extract/fractions ethyl acetate fraction exhibited the admirable antioxidant and DNA protective activities [13]. In vivo evaluation of the plant was needed to ensure its protective effects against CCl_4_ induced toxicity in testes of rat. In this regard we have assessed the activity level of antioxidant enzymes and lipid peroxidation in testes samples whereas the concentration of testosterone in serum of rat. Histopathological investigations on testes samples were also carried out to endorse the effect of various treatments.

## Methods

### Plant collection

Plants of *J. dolomiaea* were collected in 2011 from Nazar zera area of Kohistan, Pakistan. The plants were recognized by their local names and then confirmed by Dr. Mir Ajab Khan, Department of Plant Sciences, Quaid-i-Azam University, Islamabad. Voucher specimen with Accession No. 27823 was deposited at the Herbarium, Quaid-i-Azam University, Islamabad.

### Extract preparation

After collection, roots were shade dried till the complete removal of moisture and samples were made to mesh sized powder by using plant grinder and powder (5 kg) was soaked in crude methanol (10 L) for extraction for 72 h. The extraction was repeated two times with above procedure. For the purpose of filtration, Whatman No. 1 filter was used and methanol was evaporated on a rotary evaporator at 40 °C under reduced pressure to get the viscous material and fractionated on escalating polarity basis. Ethyl acetate fraction (JDEE) exhibited the most promising antioxidant abilities for various assays [13] thus was selected for in vivo evaluation against CCl_4_ induced testicular in a rat model.

### Acute toxicity studies

For acute toxicity study 18 Sprague–Dawley male rats of good health were randomly divided into six groups (3 rats in each). Animals were off feed but had open access to water 15 h prior of test samples. The control group orally received 15% DMSO in olive oil. However, rats of other groups 2–6 orally received 250, 500, 1000, 2000, and 4000 mg/kg of JDEE. General behavior of animals was noted after 120 min of treatment. Food and water were given ad libitum. Animals were screened for mortality and morbidity for 14 days [14].

### CCl_4_ induced toxicity studies in rat

For in vivo evaluation of JDEE, CCl_4_ was used to induce toxicity in testes of rats, used as an animal model. Guidelines of National Institutes of Health, Islamabad were strictly followed in order to conduct experiments effectively. The designed protocol (Bch#248) was then approved by the Ethical Committee of Quaid-i-Azam University, Islamabad, Pakistan. Animals were kept at room temperature (25 ± 3 °C) with a 12 h dark/light cycle in ordinary cages. Animals were properly fed on standard laboratory feed and water.

The rats were then left for adaptation to laboratory condition for 7 days before the commencement of experiment. The sixty day experiment was designed according to Patrick et al. [15] with minor modifications. For this purpose 42 male Sprague Dawley (*Rattus novergicus*) rats (180–200 g) were randomly divided in to 7 groups with 6 rats in each. JDEE and silymarin after dissolving in DMSO (500 mg/ml) was mixed with olive oil (1:1; *v*/v). Animals of Group I were remained untreated while of Group II were treated with the vehicle (1 ml/kg). Group III were treated (i.p.) with 1 ml/kg of CCl_4_ (1:10; *v*/v) after dissolving in olive oil and DMSO (1:1; *v*/v). The rats of Group IV were treated with CCl_4_ and co-administered with silymarin (200 mg/kg; p.o.); however, the rats of Group V and VI were treated with CCl_4_ and co-administered (p.o.) with JDEE (200 mg/kg; 400 mg/kg, respectively). Rats of Group VII were administered (p.o.) alone with 400 mg/kg of JDEE. All these treatments were given in the morning for 60 days. Rats were remained unfed for 24 h after the last treatment. Animals were euthanized after chloroform light anesthesia and dissected from ventral side. Blood was collected for testosterone measurement in the serum. Testes were excised from each animal and placed in saline solution. For histology one of the testes was stored in 10% formalin solution while the other was stored in liquid nitrogen.

### Analysis of testes homogenates

Testes samples were homogenized and mixed with 10 volume of 100 mM potassium phosphate buffer containing 1 mM EDTA (pH 7.4). Centrifugation of the homogenates was done for 30 min at 12000 *g* at 4 °C. The supernatant was collected which was used for further analyses. The total amount of soluble proteins in tissue homogenates of testes was determined by using crystalline BSA as standard [16].

### Catalase (CAT) activity

CAT activity determination was based on the process which depends on the decomposition of H_2_O_2_. The reaction mixture was prepared by the addition of 100 μl of 5.9 mM H_2_O_2_, 625 μl of 50 mM potassium phosphate buffer (pH 5.0) and 25 μl of tissue homogenate. Disappearance of H_2_O_2_ by catalase was measured in the reaction mixture at 240 nm spectrophotometrically. Absorbance change of 0.01 as units/min defines one unit CAT activity [17].

### Peroxidase (POD) activity

Chance and Maehly [17] method was used to determine POD activity in the the samples. For this the reaction mixture contained 25 μl of tissue homogenate, 25 μl of 20 mM guaiacol, 75 μl of 40 mM H_2_O_2_ and 625 μl of 50 mM potassium phosphate buffer (pH 5.0). At 470 nm change in absorbance was recorded. Change in absorbance of 0.01 as units/min defines one unit POD activity.

### Superoxide dismutase (SOD) activity

SOD activity was determined by Kakkar et al. [18] method. Tissue homogenates were first centrifuged at 1500 *g* for 10 min and then for 15 min at 10000 *g*. From the supernatant obtained a volume of 150 μl was added to the reaction mixture containing 50 μl of 186 μM phenazine methosulphate and 600 μl of 0.052 mM sodium pyrophosphate buffer (pH 7.0). In order to start the reaction 100 μl of 780 μM NADH was added and then 500 μl of glacial acetic acid was added after 1 min to stop the reaction. Absorbance of the reaction mixture was recorded at 560 nm. Finally the results obtained were expressed in units/mg protein.

### Glutathione-S-transferase (GST) activity

According to Habig et al. [19] activity of glutathione-S-transferase was based on the formation of conjugate between GSH and 1-chloro-2,4-dinitrobenzene (CDNB). The reaction mixture was prepared by the addition of 150 μl of tissue homogenate, 12.5 μl of 1 mM CDNB, 100 μl of 1 mM GSH and 720 μl of sodium phosphate buffer. At 340 nm absorbance of the reaction mixture was recorded and with molar extinction coefficient of 9.6 × 10^3^/M/cm, GST activity was determined, expressed as nM CDNB conjugate formed/min/mg protein.

### Glutathione peroxidase (GPx) activity

According to Mohandas et al. [20], NADPH was used as substrate in order to determine GPx activity. For this purpose the reaction mixture was constituted by the addition of 50 μl of tissue homogenate in 50 μl of 1 mM sodium azide, 50 μl of 1 mM EDTA, 25 μl of glutathione reductase (1 unit/ml), 25 μl of 1 mM GSH, 5 μl of 0.25 mM H_2_O_2_ and 740 μl of 0.1 M sodium phosphate buffer (pH 7.4). To initiate the reaction 50 μl of 0.2 mM NADPH was added and decline in absorbance was recorded at 340 nm at 25 °C for 20 min. Blank tubes contained only distilled water. By using molar extinction coefficient (6.22 × 10^3^/M/cm) activity of GPx was assessed and expressed as nM of NADPH oxidized/min/mg protein.

### Glutathione reductase (GR) activity

NADPH was used as substrate in order to determine GSR activity. To 50 μl of tissue homogenate, 25 μl of 1 mM oxidized glutathione, 50 μl of 0.1 mM NADPH, 50 μl of 0.5 mM EDTA and 825 μl of 0.1 M sodium phosphate buffer (pH 7.6) were added. Decomposition of NADPH was measured spectrophotometrically at 340 nm (25 °C). With the help of the molar extinction coefficient (6.22 × 10^3^/M/cm), enzymatic activity (GSR) was expressed as nM NADPH oxidized/min/mg protein [21].

### Reduced glutathione (GSH)

The method of Jollow et al. [22] was followed to measure GSH activity in the testes samples. Briefly, 500 μl of testes supernatant was mixed with 500 μl of sulfosalicylic (4%) to carry out precipitation. The reaction mixture was incubated for 1 h at 4 °C and then centrifuged at 1200 *g* for 20 min. Supernatant was collected and 33 μl of it was added to the reaction mixture containing 66 μl of 100 mM of 5,5′-dithio-bis (2-nitrobenzoic acid (DTNB) and 900 μl of 0.1 M potassium phosphate buffer (pH 7.4). The yellow colored complex was formed due to the reaction of reduced glutathione with DTNB. At 412 nm absorbance was immediately read and the GSH activity was presented by μM GSH/g tissue.

### Lipid peroxidation assay (TBARS)

The method of Ohkawa et al. [23] was used to measure the TBARS (thiobarbituric acid reactive substances) in testes samples. The reaction mixture consisted of 100 μl of tissue homogenate, 10 μl of 100 mM FeCl_3_, 100 μl of 100 mM ascorbic acid and 290 μl of sodium phosphate buffer (pH 7.4). The reaction mixture was incubated for 1 h at 37 °C in a shaking water bath at 37 °C. In order to stop the reaction 500 μl of 10% trichloro-acetic acid (TCA) was added and after an addition of 500 μl of 0.67% thiobarbituric acid (TBA) the mixture was placed in boiling water bath for 15 min. Then it was shifted on crushed ice for 5 min and centrifuged at 2500 *g* for 10 min. In order to determine the amount of TBARS formed, the absorbance of the supernatant was recorded at 535 nm. With the help of the molar extinction coefficient (1.560 × 10^5^/M/cm), lipid peroxidation activity (TBARS) was expressed as TBARS formed/min/mg tissue.

### Nitrite assay

With the help of the Griess reagent nitrite assay was carried out according to the method of Green et al. [24]. The homogenate was treated with an equal volume of (100 μl) of 0.3 M NaOH and 5% ZnSO_4._ The mixture was then centrifuged at 6400 *g* for 20 min to get the protein free supernatant. Griess reagent (1 ml) was added into the cuvette to blank the spectrophotometer. Then 20 μl of supernatant was added to the cuvette containing reagent and change in color was recorded at 540 nm. Nitrite concentration was calculated in the tissue samples by using standard curve of sodium nitrite.

### Hydrogen peroxide (H_2_O_2_) assay

The protocol of Pick and Keisari [25] was followed to assess the level of hydrogen peroxide (H_2_O_2_) in the testes samples. The oxidation of phenol red was carried out by H_2_O_2_-mediated horseradish peroxidase enzyme. The reaction mixture was prepared by the addition of 1 ml of 0.28 nM phenol red, 2.0 ml of sample homogenate, 5.5 nM dextrose, 0.05 M phosphate buffer (pH 7) and horseradish peroxidase (8.5 units) and incubated for 60 min at 37 °C. To stop the reaction 0.01 ml of 1 N NaOH was added and centrifuged at 800 *g* for 5–10 min. The absorbance of the sample was noted at 610 nm by using the reagent as a blank. The concentration of H_2_O_2_ was given as nM H_2_O_2_/min/mg tissue based on the standard curve of H_2_O_2_ oxidized phenol red.

### Analysis of testosterone in serum

The concentration of testosterone in serum was estimated through Astra Biotech kit purchased from Immunotech Company. Sensitivity of the kit is 0.2 nmol/L – 50 nmol/L. The experiment was performed according to the instruction.

### Histopathological study of testes

The testes samples were fixed in fixative sera for 3–4 h, then with the help of ascending grades of alcohol (80%, 90%, and 100%) tissues were dehydrated and finally shifted in cedar wood oil. Tissues after becoming clear were embedded in paraplast. Thin slices (3–4 μm) were prepared with the help of the microtome and then after removing wax, these were stained with hematoxylin-eosin stain, examined under microscope at 40×.

### Statistical analysis

All parametric values were expressed as means ± standard deviation (SD) of six observations. To determine the difference among various treatments one way analysis of variance was estimated by using the Statistix 8.1. Multiple comparisons among various treatments were determined by using Boneferroni post hoc comparison test. A *P* value <0.05 was considered significant.

## Results

### Effect of JDEE on antioxidant enzymes of testes

**Fig. 1:**
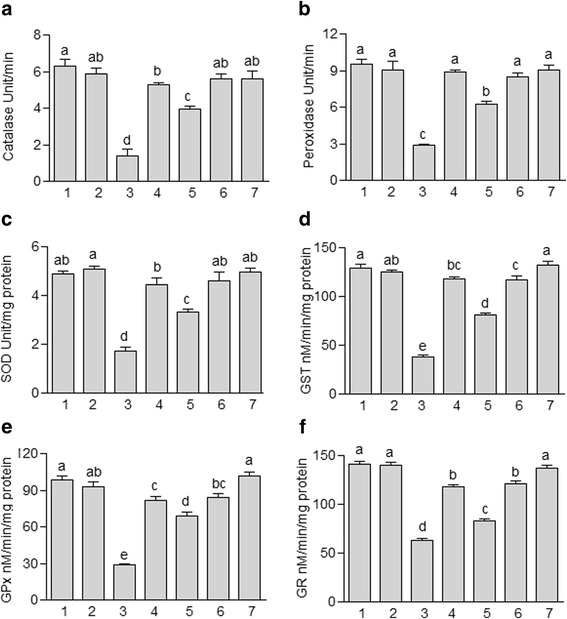
Effect of different treatments of *J. dolomiaea* ethyl acetate fraction (JDEE) on (**a**) catalase (**b**) peroxidase (**c**) superoxide dismutase (**d**) glutathione-S-transferase (**e**) glutathione peroxidase (**f**) glutathione reductase in testes of rat. (1) Control, (2) Vehicle control, (3) CCl_4_ treated control, (4) CCl_4_ + Silymarin (200 mg/kg), (5) CCl_4_ + JDEE (200 mg/kg), (6) CCl_4_ + JDEE (400 mg/kg), (7) JDEE (400 mg/kg). Bars with different letters indicate significant difference (*P* < 0.05)

In this study the activity level of CAT, POD, SOD in testes samples of CCl_4_ treated rats decreased as compared to the control group (Fig. 1). Administration of silymarin in combination with CCl_4_ ameliorated the toxic effect of CCl_4_ and increased the activity level of CAT, POD, SOD in testes samples and nonsignificant difference with the control was recorded. In case of JDEE co-treatment with CCl_4_, the activity level of CAT, POD, SOD, dose dependently, elevated in the testes samples as compared to CCl_4_ alone treated group. The higher dose of JDEE (400 mg/kg) along with CCl_4_ ameliorated the toxic effects of CCl_4_; significantly enhanced the level of CAT, POD, SOD and non significant (*P* > 0.05) difference was observed in comparison to that of the control group. However, the activity level of CAT, POD, SOD in testes of rat with JDEE (400 mg/kg) treatment alone was not changed as compared to the control group.

Activity level of GST, GPx and GR after various treatments is shown in Fig. 1. The activity level of GST, GPx and GR in testes samples treated with CCl_4_ decreased (*P* < 0.05) as comparison to the control group. Co-treatment of silymarin reference drug in combination with CCl_4_ significantly (*P* < 0.05) increased the level of these enzymes as compared to the CCl_4_ group. However, the level of these enzymes with co-treatment of silymarin was significantly (*P* < 0.05) lower as compared to the control group. In groups of CCl_4_ along with JDEE treatment activity level of GST, GPx and GR in testes samples was increased, dose dependently, as compared to the CCl_4_ group. The higher dose of JDEE (400 mg/kg) produced similar protective effects (*P* > 0.05) for these enzymes to that of the silymarin co-treated group. Rats when orally treated with JDEE (400 mg/kg) alone did not alter the activity level of these enzymes as compared to the control group.

### Effect of JDEE on biochemical profile of testes

**Fig. 2:**
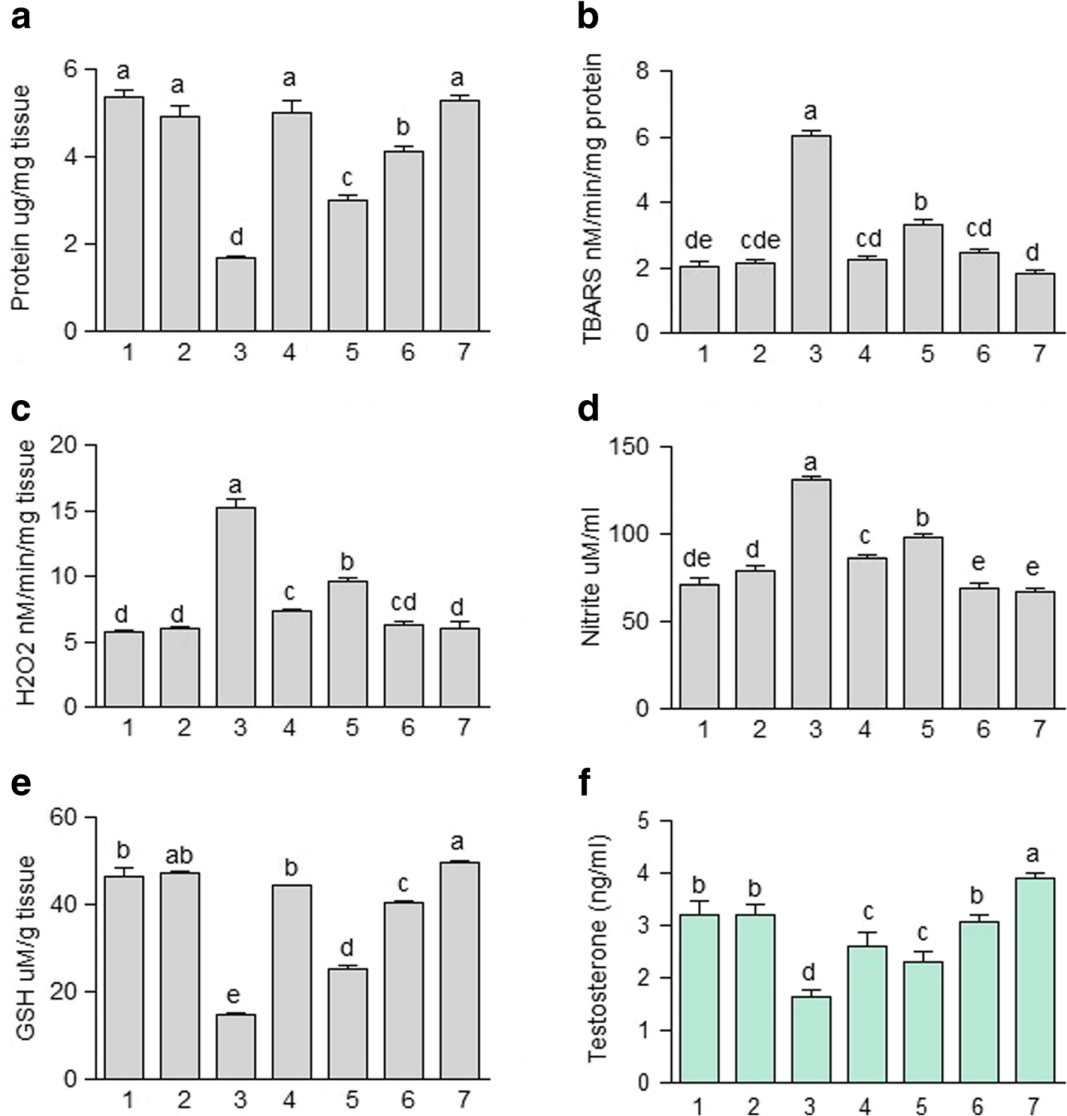
Effect of different treatments of *J. dolomiaea* ethyl acetate fraction (JDEE) on (**a**) protein (**b**) thiobarbituric acid reactive substances (**c**) hydrogen peroxide (**d**) nitrite (**e**) glutathione in testes (**f**) testosterone in serum of rat. (1) Control, (2) Vehicle control, (3) CCl_4_ treated control, (4) CCl_4_ + Silymarin (200 mg/kg), (5) CCl_4_ + JDEE (200 mg/kg), (6) CCl_4_ + JDEE (400 mg/kg), (7) JDEE (400 mg/kg). Bars with different letters indicate significant difference (*P* < 0.05)

The profile of testes protein, TBARS, H_2_O_2_ and nitrite content of different groups is shown in Fig. 2. It is apparent from Fig. 2 that concentration of protein in testes of rat was significantly (*P* < 0.05) decreased in CCl_4_ treated group as compared to the control group. Co-administration of JDEE along with CCl_4_, dose dependently, increased the level of protein in testes samples. However, complete protection was not attained even at the highest dose of JDEE (400 mg/kg) and significantly lower level of protein was recorded in comparison to that of the control group. Rats treated with CCl_4_ in combination with silymarin exhibited the protein level similar (*P* > 0.05) to that of the control group. Treatment of JDEE (400 mg/kg) alone to rats did not change the level of protein as compared to the control group.

The level of TBARS, H_2_O_2_ and nitrite was significantly (*P* < 0.05) elevated in CCl_4_ treated group as compared to the control group (Fig. 2). Concentration of TBARS, H_2_O_2_ and nitrite was diminished, dose dependently, and at higher dose of JDEE (400 mg/kg) co-administered with CCl_4_ similar level of these parameters to that of the control group was observed. However, co-administration of JDEE at higher dose of 400 mg/kg showed more protection in terms of nitrite content than the reference silymarin treated group.

The level of GSH in testes samples of various groups is shown in Fig. 2. The results indicated that administration of CCl_4_ to rats cause toxicity and depleted the level of GSH in testes as compared to the control group. Co-treatment of reference drug silymarin with CCl_4_ was able to maintain the level of GSH in testes sample similar to that of the control group. On the other hand co-administration of JDEE ameliorated the toxicity of CCl_4_ and increased the level of GSH in testes samples in a dose dependent manner. However, the lower and the higher dose did not totally ameliorated the toxicity of CCl_4_ thus the level of GSH obtained in testes samples was significantly (*P* < 0.05) lower as compared to the control group. Further, administration of JDEE (400 mg/kg) alone to rats significantly (*P* < 0.05) increased the level of GSH as compared to the control group.

### Effect of JDEE on testosterone

Level of testosterone in serum of different groups is displayed in Fig. 2. Treatment of CCl_4_ exhibited significant (*P* < 0.05) reduction in testosterone level of serum as compared to the control group of rat. Co-administration of silymarin as a reference drug in this experiment showed an elevation in the level of testosterone in comparison to that of the CCl_4_ treated group but its level was significantly (*P* < 0.05) less to that of the control group. Treatment of JDEE in combination with CCl_4_, dose dependently, increased the level of testosterone and at the higher dose (400 mg/kg) nonsignificant (*P* > 0.05) difference was observed in comparison to that of the control group. However, when rats were treated with JDEE (400 mg/kg) alone, significantly (*P* < 0.05) higher level of testosterone was recorded in comparison to that of the control group.

### Protective role of JDEE on histoarchitecture of testes

**Fig. 3:**
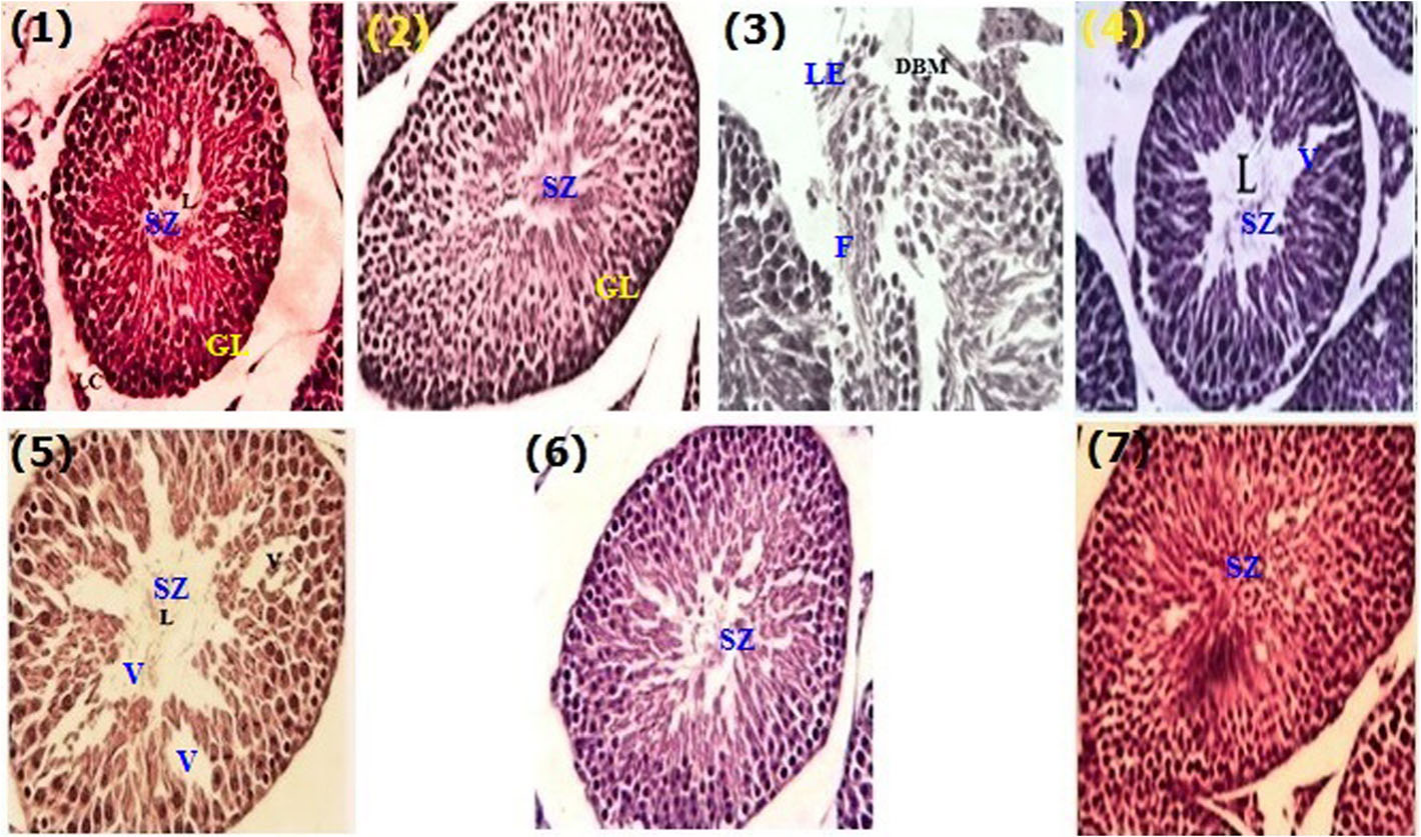
40 ×; Microphotographs of testes histology of different groups after *J. dolomiaea* ethyl acetate fraction (JDEE) treatment (H & E staining). **1** Control, **2** Vehicle control, **3** CCl_4_ treated control, **4** CCl_4_ + Silymarin (200 mg/kg), **5** CCl_4_ + JDEE (200 mg/kg), **6** CCl_4_ + JDEE (400 mg/kg), **7** JDEE (400 mg/kg). SZ; spermatozoa, F; fibers, LE; loss of epithelium, V; vacuole

Effect of JDEE on testes histoarchitecture in different groups is illustrated in Fig. 3. The control group and the negative control group did not exhibit any alteration and displayed normal architecture of seminiferous tubules normal developmental stages and concentration sperms in the seminiferous tubules. CCl_4_ treatment resulted in rupturing, displacement and alteration in shapes of seminiferous tubules. Vacuolization of germinal epithelium and prominent decrease in germ cells was also recorded. Silymarin co-treatment showed marked protection in terms of morphology of the seminiferous tubules and the density of germ cells. In JDEE co-treated groups ameliorative effects against CCl_4_ induced toxicity were observed on the architecture of the seminiferous tubules in a dose dependent fashion. JDEE at low dose illustrated some vacuoles in seminiferous epithelium which were absent at the higher dose (400 mg/kg). JDEE when treated alone demonstrated high density of germinal cells and sperms in the seminiferous tubules.

## Discussion

Development of spermatozoa from spermatogonial stem cells is regulated by various hormones and this whole process in controlled by the hypothalamic-pituitary-testicular axis. Any minor disturbance either imposed internally or from the external environment such as chemicals, lead to fertility problems in males. Accumulation of ROS in testis induces hypogonadism [3]. Exposure of rats to CCl_4_ prompted significant reduction in CAT, POD, SOD and GPx profile, exhausted the GSH level and elevated TBARS in testes [2–4]. Higher production of H_2_O_2_ and nitrite in testes samples on account of CCl_4_ treatment suppresses the antioxidant defense while enhances the cellular injuries [2–4]. JDEE constituted significant amount of total flavonoids 807.0 ± 7.2 mg rutin equivalent/g of JDEE. It can be argued that protection provided against oxidative stress by JDEE can be attributed by the presence of flavonoids and other antioxidant constituents. As JDEE interfere with the oxidation process by scavenging, reducing and chelation of free radical [13] that is reflected in decrease of TBARS and increase in total antioxidant capacity. It is suggested that the presence of flavonoids in JDEE might play a major role in treating or retarding oxidative stress related fertility disorders; and can improve fertility rate in men.

Enhanced level of GSH in the rats treated with JDEE (400 mg/kg) alone was recorded in this study. As GSH regulate the level of antioxidant enzymes, it may be intriguing that JDEE can be helpful to control the oxidative status and consequently the libido and erectile function in males. Increase in GSH level with juice and methanol extract of pomegranate peel has been determined in rat [26]. In another study use of pomegranate juice increased the GSH in testis of rat [27].

CCl_4_ impelled testes damages have been connected with high nitrite generation in this study. Peroxynitrite anions have been created by the response of nitric oxide and superoxide anion. These peroxynitrite anions oxidize biomolecules, which finally prompts lipid peroxidation [3, 4]. Free radical scavenging capability of JDEE may be the reason in defensive impact against CCl_4_ harmfulness. JDEE hold the active constituents (flavonoids, terpenoids, saponins) which directly or indirectly scavenge the oxidative harm to various cells and organs while normalizing their oxidative status [3, 4]. Deterioration of seminiferous tubules and germ cells, interstitial in part was vanished and replaced by fibroblast and inflammatory cells were recorded in testes of CCl_4_ treated group of this study. Presence of flavonoids in JDEE could be included in ameliorating the impacts of CCl_4_ prompted injuries and correspondingly close to typical histology of testis. Similar histopathological alterations were recorded while assessing the defensive impact of various extracts of plants on testes against CCl_4_ induced toxicity [3, 4]. Administration of JDEE alone to rats caused the increase in thickness of germinal layer of seminiferous tubules. Increase in the diameter of seminiferous tubules and increase in germinal thickness has been demonstrated in rat with pomegranate juice and alcoholic extract of *Nigella sativa* seeds [27, 28].

In the present study, testosterone concentration was quantified and was observed altogether low in correlation to that of the standard control groups. Treatment of animals with CCl_4_ in combination with JDEE ameliorated the harmful impacts of CCl_4_ and the level of testosterone was increased, in a dose dependent way. JDEE hold the constituents (flavonoids, terpenoids and saponins) which directly or indirectly ameliorate the oxidative harm to distinctive cells and organs. Protective effect of plants against the CCl_4_ induced testicular toxicity have been reported [3, 4]. However, the administration of JDEE (400 mg/kg) alone to rats significantly enhanced the level of testosterone in serum as compared to the control group. Presence of saponins in the JDEE might be responsible for the release of pituitary LH while the flavonoids might have been implicated in the synthesis of androgens. Higher testosterone level with pomegranate juice and alcoholic extract of *Nigella sativa* seeds has been determined in rat [27, 28]. It is suggested that the enhanced level of testosterone with JDEE in male rats might be beneficial to increase libido and sexual function of human being.

## Conclusions

The results of the present investigation suggested the antioxidant effects of the JDEE against the oxidative stress induced with CCl_4_ in testes of male rates. Diminished level of CAT, POD, SOD, GPx, GST, GR and GSH while enhanced level of TBARS, nitrite and H_2_O_2_ in testes samples of male rats was reversed back to the control, dose dependently, by the co-administration of JDEE. The altered histopathological changes induced with CCl_4_ were also diminished with co-treatment of JDEE. However, an admirable effect of JDEE alone to male rats was the enhanced level of GSH in testes and testosterone in serum samples. The thickness of the germinal epithelium was also increased. These results suggested the evaluation of JDEE for sexual behavior.

## Abbreviations

CAT: Catalase
CCl_4_: Carbon tetrachloride
GPx: Glutathione peroxidase
GR: Glutathione reductase
GSH: Glutathione
GST: Glutathione-S-transferase
H_2_O_2_: Hydrogen peroxide
JDEE: *Jurenia dolomiaea* ethyl acetate fraction of crude methanol extract
POD: Peroxidase
SOD: Superoxide dismutase
TBARS: Thiobarbituric acid reactive substances
